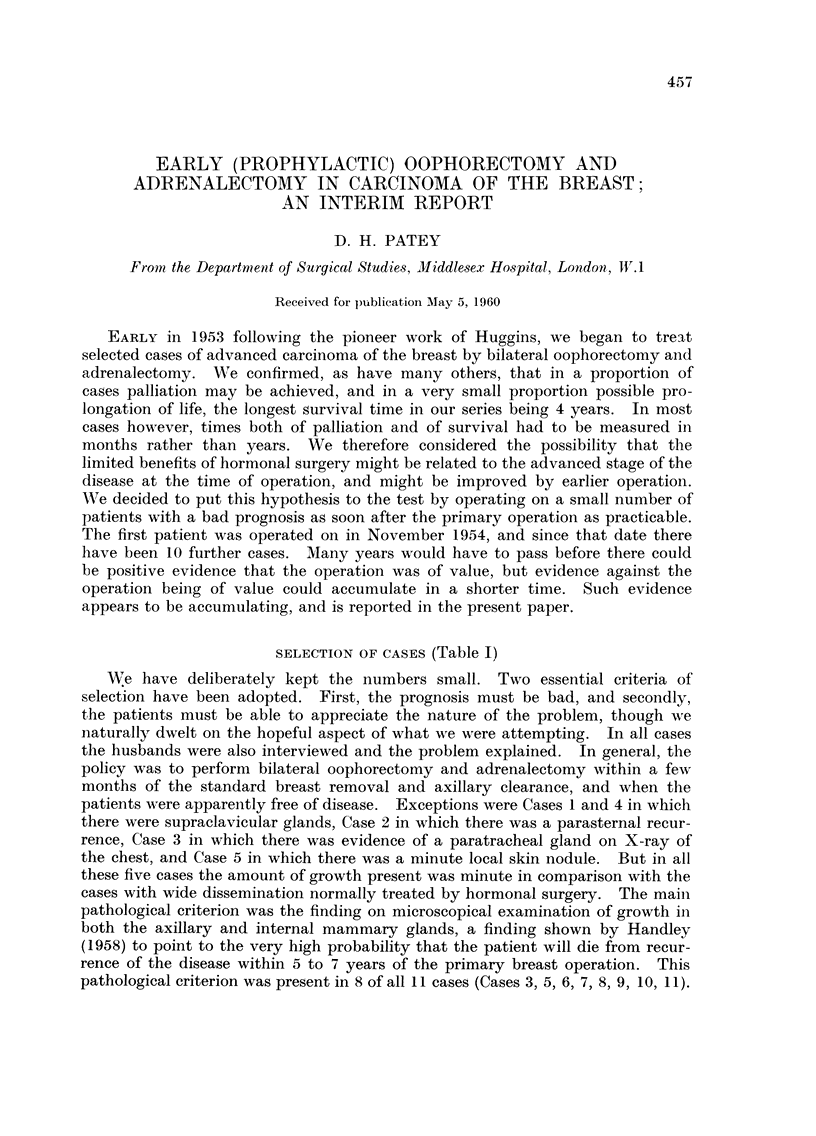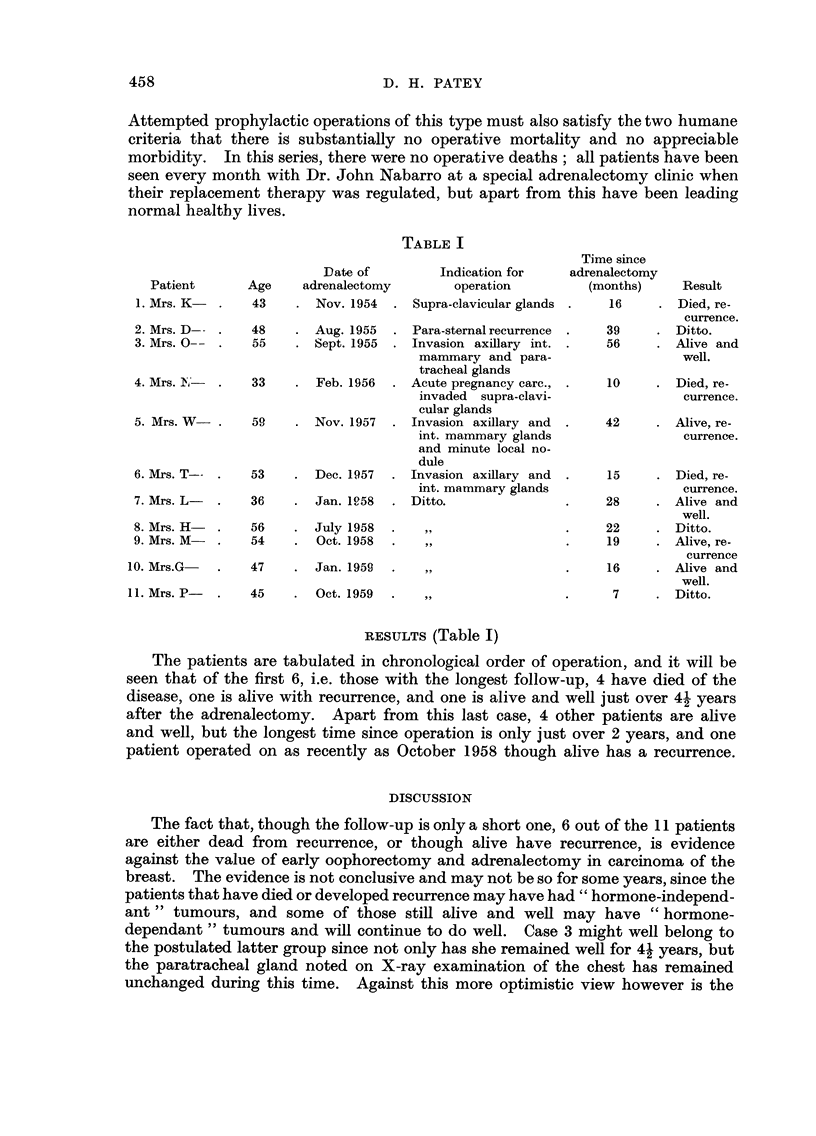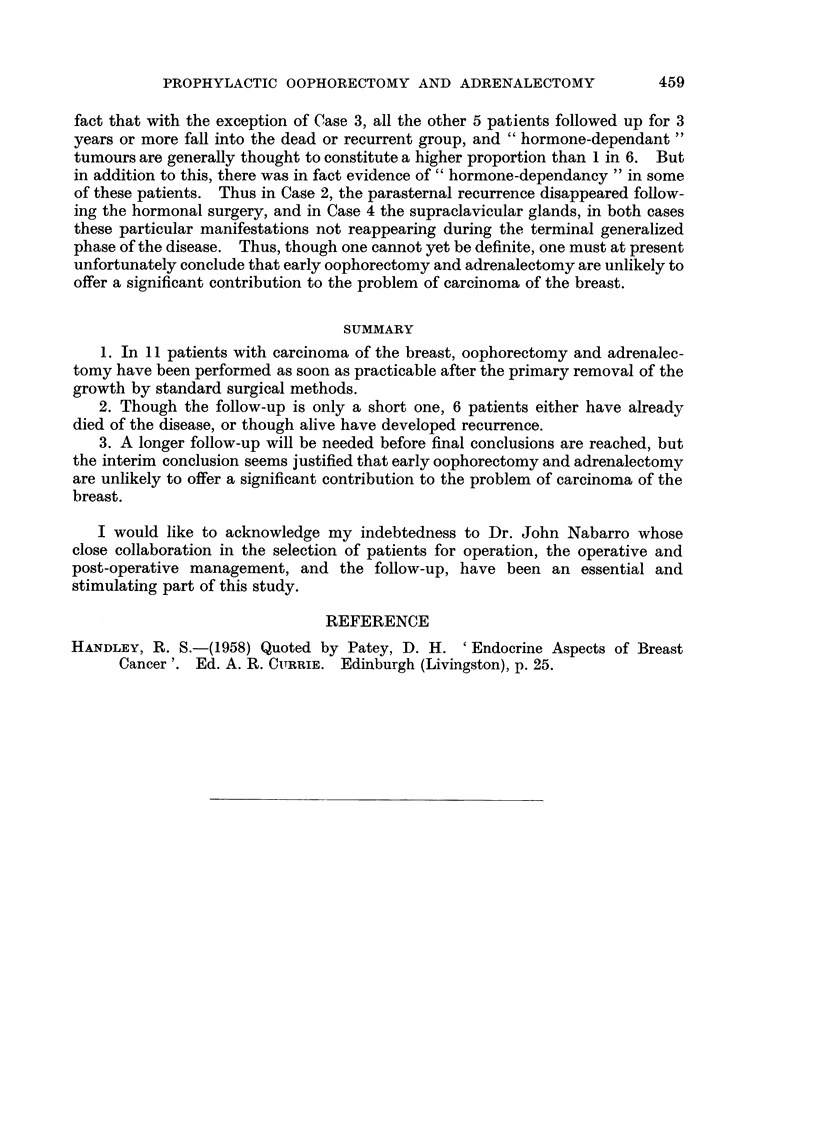# Early (Prophylactic) Oophorectomy and Adrenalectomy in Carcinoma of the Breast; An Interim Report

**DOI:** 10.1038/bjc.1960.49

**Published:** 1960-09

**Authors:** D. H. Patey


### EARLY (PROPHYLACTIC) OOPHORECTOMY AND ADRENALECTOMY IN CARCINOMA OF THE BREAST; AN INTERIM REPORT

D. H. PATEY

*From the Department of Surgical Studies, Middlesex Hospital, London, W.1*

Received for publication May 5, 1960

EARLY in 1953 following the pioneer work of Huggins, we began to treat selected cases of advanced carcinoma of the breast by bilateral oophorectomy and adrenalectomy. We confirmed, as have many others, that in a proportion of cases palliation may be achieved, and in a very small proportion possible prolongation of life, the longest survival time in our series being 4 years. In most cases however, times both of palliation and of survival had to be measured in months rather than years. We therefore considered the possibility that the limited benefits of hormonal surgery might be related to the advanced stage of the disease at the time of operation, and might be improved by earlier operation. We decided to put this hypothesis to the test by operating on a small number of patients with a bad prognosis as soon after the primary operation as practicable. The first patient was operated on in November 1954, and since that date there have been 10 further cases. Many years would have to pass before there could be positive evidence that the operation was of value, but evidence against the operation being of value could accumulate in a shorter time. Such evidence appears to be accumulating, and is reported in the present paper.

### SELECTION OF CASES (Table I)

We have deliberately kept the numbers small. Two essential criteria of selection have been adopted. First, the prognosis must be bad, and secondly, the patients must be able to appreciate the nature of the problem, though we naturally dwelt on the hopeful aspect of what we were attempting. In all cases the husbands were also interviewed and the problem explained. In general, the policy was to perform bilateral oophorectomy and adrenalectomy within a few months of the standard breast removal and axillary clearance, and when the patients were apparently free of disease. Exceptions were Cases 1 and 4 in which there were supraclavicular glands, Case 2 in which there was a parasternal recurrence, Case 3 in which there was evidence of a paratracheal gland on X-ray of the chest, and Case 5 in which there was a minute local skin nodule. But in all these five cases the amount of growth present was minute in comparison with the cases with wide dissemination normally treated by hormonal surgery. The main pathological criterion was the finding on microscopical examination of growth in both the axillary and internal mammary glands, a finding shown by Handley (1958) to point to the very high probability that the patient will die from recurrence of the disease within 5 to 7 years of the primary breast operation. This pathological criterion was present in 8 of all 11 cases (Cases 3, 5, 6, 7, 8, 9, 10, 11).

Attempted prophylactic operations of this type must also satisfy the two humane criteria that there is substantially no operative mortality and no appreciable morbidity. In this series, there were no operative deaths ; all patients have been seen every month with Dr. John Nabarro at a special adrenalectomy clinic when their replacement therapy was regulated, but apart from this have been leading normal healthy lives.

TABLE I

| Patient | Age | Date of adrenalectomy | Indication for operation | Time since adrenalectomy (months) | Result |
|---|---|---|---|---|---|
| 1. Mrs. K— | 43 | Nov. 1954 | Supra-clavicular glands | 16 | Died, recurrence. |
| 2. Mrs. D— | 48 | Aug. 1955 | Para-sternal recurrence | 39 | Ditto. |
| 3. Mrs. O— | 55 | Sept. 1955 | Invasion axillary int. mammary and paratracheal glands | 56 | Alive and well. |
| 4. Mrs. N— | 33 | Feb. 1956 | Acute pregnancy carc., invaded supra-clavicular glands | 10 | Died, recurrence. |
| 5. Mrs. W— | 59 | Nov. 1957 | Invasion axillary and int. mammary glands and minute local nodule | 42 | Alive, recurrence. |
| 6. Mrs. T— | 53 | Dec. 1957 | Invasion axillary and int. mammary glands | 15 | Died, recurrence. |
| 7. Mrs. L— | 36 | Jan. 1958 | Ditto. | 28 | Alive and well. |
| 8. Mrs. H— | 56 | July 1958 | ,, | 22 | Ditto. |
| 9. Mrs. M— | 54 | Oct. 1958 | ,, | 19 | Alive, recurrence |
| 10. Mrs.G— | 47 | Jan. 1959 | ,, | 16 | Alive and well. |
| 11. Mrs. P— | 45 | Oct. 1959 | ,, | 7 | Ditto. |

RESULTS (Table I)

The patients are tabulated in chronological order of operation, and it will be seen that of the first 6, i.e. those with the longest follow-up, 4 have died of the disease, one is alive with recurrence, and one is alive and well just over $4\frac{1}{2}$ years after the adrenalectomy. Apart from this last case, 4 other patients are alive and well, but the longest time since operation is only just over 2 years, and one patient operated on as recently as October 1958 though alive has a recurrence.

DISCUSSION

The fact that, though the follow-up is only a short one, 6 out of the 11 patients are either dead from recurrence, or though alive have recurrence, is evidence against the value of early oophorectomy and adrenalectomy in carcinoma of the breast. The evidence is not conclusive and may not be so for some years, since the patients that have died or developed recurrence may have had " hormone-independant " tumours, and some of those still alive and well may have " hormone-dependant " tumours and will continue to do well. Case 3 might well belong to the postulated latter group since not only has she remained well for $4\frac{1}{2}$ years, but the paratracheal gland noted on X-ray examination of the chest has remained unchanged during this time. Against this more optimistic view however is the fact that with the exception of Case 3, all the other 5 patients followed up for 3 years or more fall into the dead or recurrent group, and " hormone-dependant " tumours are generally thought to constitute a higher proportion than 1 in 6. But in addition to this, there was in fact evidence of " hormone-dependancy " in some of these patients. Thus in Case 2, the parasternal recurrence disappeared following the hormonal surgery, and in Case 4 the supraclavicular glands, in both cases these particular manifestations not reappearing during the terminal generalized phase of the disease. Thus, though one cannot yet be definite, one must at present unfortunately conclude that early oophorectomy and adrenalectomy are unlikely to offer a significant contribution to the problem of carcinoma of the breast.

### SUMMARY

1. In 11 patients with carcinoma of the breast, oophorectomy and adrenalectomy have been performed as soon as practicable after the primary removal of the growth by standard surgical methods.
2. Though the follow-up is only a short one, 6 patients either have already died of the disease, or though alive have developed recurrence.
3. A longer follow-up will be needed before final conclusions are reached, but the interim conclusion seems justified that early oophorectomy and adrenalectomy are unlikely to offer a significant contribution to the problem of carcinoma of the breast.

I would like to acknowledge my indebtedness to Dr. John Nabarro whose close collaboration in the selection of patients for operation, the operative and post-operative management, and the follow-up, have been an essential and stimulating part of this study.

### REFERENCE

HANDLEY, R. S.—(1958) Quoted by Patey, D. H. 'Endocrine Aspects of Breast Cancer'. Ed. A. R. CURRIE. Edinburgh (Livingston), p. 25.